# Multi-Objective Welding-Parameter Optimization Using Overlaid Contour Plots and the Butterfly Optimization Algorithm

**DOI:** 10.3390/ma15134507

**Published:** 2022-06-27

**Authors:** Rehan Waheed, Hasan Aftab Saeed, Bilal Anjum Ahmed

**Affiliations:** Department of Mechanical Engineering (CEME), National University of Sciences and Technology (NUST), Sector H-12, Islamabad 4600, Pakistan; hasan.saeed@ceme.nust.edu.pk (H.A.S.); bilal.anjum@ceme.nust.edu.pk (B.A.A.)

**Keywords:** BOA, multi-objective optimization, residual stresses, response surface method, welding distortion

## Abstract

Distortion and residual stress are two unwelcome byproducts of welding. The former diminishes the dimensional accuracy while the latter unfavorably affects the fatigue resistance of the components being joined. The present study is a multi-objective optimization aimed at minimizing both the welding-induced residual stress as well as distortion. Current, voltage, and welding speed were the welding parameters selected. It was observed that the parameters that minimize distortion were substantially different from those that minimized the residual stress. That is, enhancing dimensional accuracy by minimizing distortion results in an intensification of residual stresses. A compromise between the two objectives was therefore necessary. The contour plots produced from the response surfaces of the two objectives were overlaid to find a region with feasible parameters for both. This feasible region was used as the domain wherein to apply the novel butterfly optimization algorithm (BOA). This is the first instance of the application of the BOA to a multi-objective welding problem. Weld simulation and a confirmatory experiment based on the optimum weld parameters thus obtained corroborate the efficacy of the framework.

## 1. Introduction

In the welding process, the parts being produced experience a thermal gradient. The temperature of the material under the welding torch is at or above the melting point of the base metal. In the same base metal, some regions are at ambient or room temperature, while others are in between the solidus and the ambient temperature. This temperature difference produces shrinkage forces in the base metal larger than the yield point of the material. These shrinkage forces during the heating and cooling cycle of the welding process produce distortion and locked-in stresses or residual stresses. The distortion and residual stresses both need to be minimized. The distortion produced during welding perturbs the dimensional requirements of the part being manufactured and causes fitment problems during assembly. The residual stresses need to be avoided for parts under fluctuating or cyclic loading. Parts of automobile frame structures, rotating machinery, airframes, and ship structures are all subjected to fatigue loading during their useful life. In all of these and similar cases, welding distortion and residual stress need to be addressed concurrently. The factors that play a vital role in producing welding distortion and residual stress need to be examined and a multi-objective optimization study is required to find the optimum values of the parameters that produce minimum distortion and residual stress in the welded structures. The experimental procedures for performing multiple test runs are expensive; therefore, welding simulations through FEA are utilized with the design of experiments (DOE) approach to find the optimum ranges of values. The response surface method (RSM) is a useful tool for this purpose. In the present work, contour plots of welding-induced distortion and residual stresses were produced from response surfaces. These contour plots were overlaid to find a feasible region of optimum weld parameters. From this feasible region is derived the solution domain for BOA optimization. The optimum welding process parameters were obtained after multiple iterations of the BOA.

## 2. Literature Review

In the past, the response surface method has been used as a tool to optimize the weld parameters. Prasada et al. [1] employed the response surface method to optimize the ultimate strength of Inconel sheets. They used a central composite rotatable design matrix and checked the influence of peak current, back current, pulse, and pulse width on the ultimate tensile strength of the base metal. Srivastava and Garg [2] used the response surface method with a Box–Behnken design. The responses they studied were the weld bead width, bead height, and the depth of penetration. Vasantharaja and Vasudevan [3] optimized the activated tungsten inert gas (TIG) welding process parameters using the response surface method. In their research, they applied the desirability approach for the optimization of the weld process parameters. Vidyarthi et al. [4] optimized the weld process parameters (i.e., welding current, speed, and flux coating density). They used the response surface method with a central composite design and studied the response of bead width, depth to width ratio of bead, weld fusion zone area, and depth of penetration. Lai et al. [5] applied the response surface method for the optimization of resistance spot welding. The response parameters they selected for the study were the electrode diameter and the effect on the electrode during the cooling process. Their study provided a useful technique for the design of resistance spot welding electrodes. Korra et al. [6] optimized the activated TIG welding process parameters (i.e., welding current, speed, and arc gap) by employing the response surface method with a central composite design. They studied the response of the process parameters on various weld bead geometry aspects and used the desirability approach for multi-objective optimization. Joseph et al. [7] used the Taguchi method for the design of experiments. In their work, they used the response surface method as a tool to develop a mathematical relation between the weld process parameters through regression analysis. This mathematical relation was used by them for further optimization of the welding process through the genetic algorithm (GA). Waheed et al. [8] used the response surface method and artificial intelligence to optimize welding induced distortion. Gunaraj and Murugan [9] used the response surface method for the optimization of weld process parameters for submerged arc welding. They used a central composite design that is rotatable and used the welding parameters (i.e., speed, arc voltage, wire feed rate, and nozzle-to-plate distance) to optimize the quality of the weld. The effect of welding residual stress during cyclic loading has been studied by many researchers. The control and optimization of residual stress are essential for welded structures under fatigue loading. Mochizuki [10] examined the problem of the minimization of the residual stress produced during welding. He concluded that the welding residual stress should be controlled during the welding process rather than relying on the post-weld heat treatment procedures.

Lee and Kyong [11] studied the fatigue crack growth rate under welding residual stress. They calculated the stress intensity factor in the presence of residual stress and used the linear elastic fracture mechanics (LEFM) technique to predict crack growth under fatigue loading. Hensel et al. [12] studied the fracture resistance of welded structures under fatigue loading. They concluded that the welding residual stress significantly affected the overall fatigue strength, crack growth rate, and fatigue life of the welded part. Farajian [13] observed in his work that weld residual stresses of magnitude equal to yield strength were present in large-welded structures. He also observed that the residual stresses present at the weld centerline were of higher magnitude than the stresses present near the toe of the weld bead. However, since the crack initiation usually starts from the toe of the weld bead during cyclic loading, this area also needs to be considered. Cui et al. [14] studied the deck-to-rib stresses in automobile bodies. The effect of stresses produced due to vehicle movement in the presence of residual stresses was the main interest of their work. They concluded that the welding residual stresses had a marked negative effect on the overall fatigue resistance. Chang [15] studied the softening of high tensile residual stresses through heat treatment procedures. He showed that high tensile stresses can be changed to compressive stresses by ultrasonic impact treatment. Barsoum and Barsoum [16] simulated fatigue crack propagation in the presence of welding residual stresses. First, they found the welding residual stresses through the finite element method (FEM) simulation. The welding residual stresses were mapped in the next simulation as an input load and the LEFM technique was then employed to predict the propagation of crack in the presence of weld residual stresses. In another study, Barsoum [17] studied the weld residual stresses near the weld root and toe in plate-to-tube joints. He performed a 2D FEM analysis and verified the simulated results with experimental data. Caruso and Imbrogno [18] performed the finite element modeling of AISI 441 steel plates and developed a user subroutine to predict the grain size variation and hardness of the steel plates. Murat and Ozler [19] developed a finite element model of friction stir welding. They predicted that the ratio of tool rotational speed and tool feed is critical for avoiding defects in friction stir welding. Moslemi et al. [20] developed a systematic procedure for calibrating heat source parameters before simulating the welding process for GMAW welding. Zhang and Dong [21] studied the possibility of brittle fracture in welded structures. They observed how residual stresses could decrease the plastic deformation capability of metals, thus decreasing the fatigue life of the welded structures.

Narwadkar and Bhosle [22] studied the angular distortion produced in the welded structures. They used the design of experiments approach. They observed the effect of welding current, voltage, and gas flow rate on the angular distortion of welded parts. Zhang et al. [23] used FEM simulations to observe the overall distortion in a large vacuum vessel, cutting down the cost of constructing a prototype of the vessel. Lorza et al. [24] built a thermomechanical model to simulate the TIG welding process. They observed the effect of welding voltage, current, speed, and torch parameters on the distortion produced during welding. Chen et al. [25] studied the distortion produced during welding in panel structures with stiffeners. The fillet joint configuration was simulated using FEM. The weld parameters of the welding current, voltage, and speed were used as the governing factors to control distortion. Additionally included in the study was the effect of the welding sequence. Multi-objective studies were adopted by several researchers to optimize the different weld responses. Rong et al. [26] optimized the longitudinal residual stress and transverse tensile stresses. Romero-Hdz et al. [27] used the GA for their multi-objective optimization of welding induced residual stresses and distortion. They used FE simulations to calculate the weld distortion and residual stresses for their study. Shao et al. [28], in a similar study, used multi-objective particle swarm optimization (MOPSO) to optimize the welding residual stress and distortion. They used welding current, speed, and voltage as the main parameters that affected the objective function.

The butterfly optimization algorithm (BOA) is a nature-inspired algorithm proposed by Arora and Singh [29]. They demonstrated the efficacy of the BOA over other nature-inspired metaheuristics by solving three classical benchmark engineering problems. They compared BOA with other nature-based optimization techniques such as artificial bee colony, cuckoo search, differential evolution, firefly algorithm, genetic algorithm, particle swarm, and the modified butterfly optimization algorithm. They found the BOA to be more efficient than the other metaheuristic algorithms. Yildiz et al. [30] applied the BOA to obtain the optimum shape of automobile suspension components, achieving a weight reduction of 32.9%. In previous research, the response surface method was used to formulate the objective function, but its outcome (i.e., the contour plots) was not utilized in the optimization process. In the present research, the boundaries of the solution domain were constrained through overlaid contour plots, which shrinks the solution domain. This helps in the implementation of BOA as a multi-objective optimization technique to find the optimum residual stress and welding induced distortion. The BOA has thereby been further enhanced to be used as a multi-objective optimization technique to obtain optimum weld parameters that produce minimum welding-induced distortion and residual stresses in the welded structures. This is the first instance of the application of the BOA to a multi-objective welding problem.

## 3. Methods

**Figure 1:**
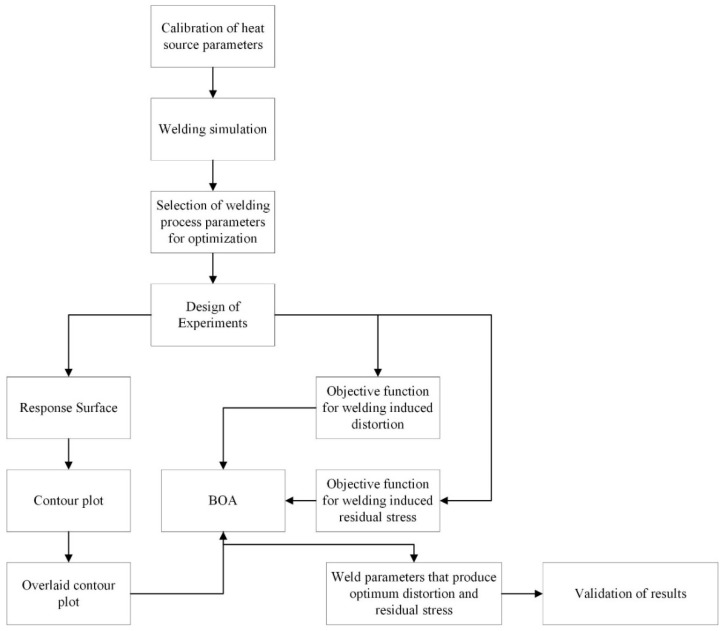
The framework of the multi-objective optimization study.

The research methods used in this study and their connection with each other are shown in Figure 1. First, a welding simulation was performed using temperature dependent material properties on a 2 mm thick ASTM A36 steel plate butt joint. The finite element analysis of the welding process requires the exact amount of heat to be applied on the base plate to obtain accurate results. For this purpose, the heat source needs to be modeled carefully. In the next step, welding process parameters that affect welding induced distortion and residual stress were identified. These parameters need to be modified to obtain the optimum values of welding induced distortion and residual stress. To observe the results of varying process parameters, a number of welding simulations are required. The DOE approach is used to minimize the number of experiments. Response surfaces and contour plots are generated as an outcome of DOE application. In addition, regression analysis is performed and the responses (i.e., the welding induced distortion and residual stress) are formulated in the form of equations. These equations and the contour plots are used to run the final optimization using BOA. The two equations serve as objective functions while the solution domain in BOA is defined by the overlaid contour plots. To validate the results of this multi-objective optimization, a welding simulation was performed using the optimum welding process parameters. Finally, a test sample was prepared to compare the results of the welding simulation with the actual values. The methods outlined above are further explained in the following subsections.

### 3.1. Welding Simulation

**Figure 2:**
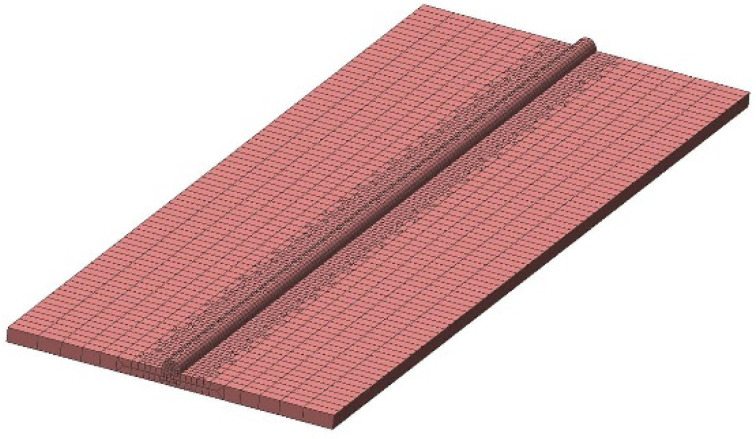
The FE model of the butt joint.

In this work, 2 mm thick ASTM A36 steel plates in a butt joint configuration were considered. To simulate the welding process, an FEM model of the base metal and the weld metal were prepared. The mesh of the FEM model is shown in Figure 2. The base material and filler material for welding process was the same (i.e., ASTM A36). The chemical composition of the base and filler material were taken from the work of Sajid and Kiran [31], as given in Table 1.

**Figure 3:**
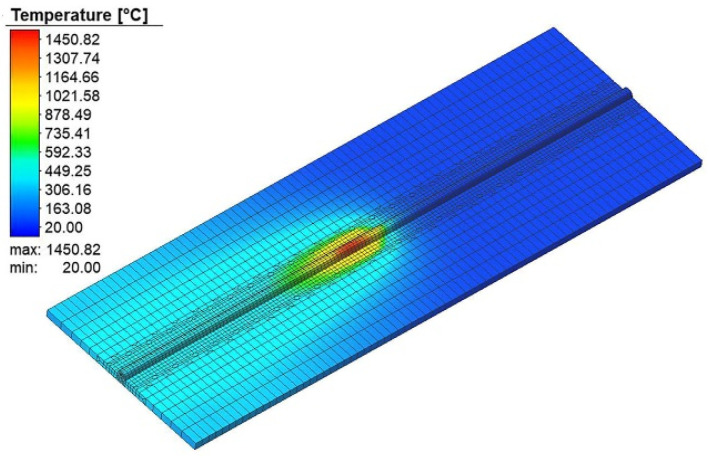
The temperature profile during the weld simulation.

A couple-field thermomechanical analysis was used to simulate the welding process. The welding simulation was performed on ANSYS. A moving heat source was developed through a user sub-routine to provide heat input to the weld area. The heat was provided through a double ellipsoidal heat source as proposed by Goldak et al. [32]. The value set for the double ellipsoidal heat source parameters is given in Table 2. In thin steel plates, the change in the color of the steel due to the temperature rise is visible after the welding process. This phenomenon helps in adjusting the parameters of the double ellipsoidal heat source to match the temperature profile of the base material. The temperature profile during weld simulation is shown in Figure 3.

The density of elements in the heat-affected zone was increased by partitioning and mapped meshing. The mesh shown in Figure 2 was hex-dominant with couple-field elements to take up heat and stress simultaneously. The numerical setting included the application of load and boundary conditions. In the welding simulation, load is the amount of heat supplied through the welding torch. To apply thermal load at the element level, a new coordinate system was defined at the starting point of the welding process. This coordinate system was moved in each time step of analysis. The coordinates of each element in the double ellipsoid region were found through a user subroutine. Heat was applied to each element in this region. As the coordinate system moved forward in the next analysis step, heat was applied to new elements. The movement of the coordinate system was linked to the welding speed. The time step during the welding process was 0.5 s. After welding, the cooling phase started. In the cooling phase, the time step-size was increased exponentially to decrease the computational cost. To obtain the distortion and residual stress, a multilinear isotropic model was used. The stresses at the melting point temperature were ignored to avoid excessive plastic strains. These plastic strains pose difficulties in the convergence of solutions; however, their impact on the accuracy of results was negligible.

Welding induced residual stress and distortion were caused by shrinkage forces generated as a result of nonuniform cooling and heating cycles. The heat generated in the welding process has a direct bearing on these shrinkage forces. The heat generated per unit length is given in Equation (1).

(1) $$Welding\;heat\;per\;unit\;length=\frac{\;Current\times Voltage}{Welding\;speed}\;$$

It is evident from Equation (1) that the current, voltage, and welding speed are the three parameters that contribute to heat input in the welding process, and are therefore intimately linked with the phenomena of residual stress and distortion. Therefore, the input parameters in the present work were the welding current, voltage, and speed. All other factors were kept constant in the thermomechanical analysis.

### 3.2. Response Surface

The response surface method is a useful tool to observe the response of a system to multiple influencing factors. It is a graphical tool to represent the response of two factors at a time in the 3D plot. If three factors are under study, then three response surfaces are required to represent their effect. In optimization problems, the objective typically is to find the maximum or minimum of a function. If a graph of objective function is plotted, then the trend of that function can be observed. The expected response E(y) is represented by Equation (2).

(2) $$\;\;E\left(y\right)=f\left(x_{1},x_{2}\;\right)=\eta \;\;$$

In Equation (2), η is the response surface for factors $x_{1}$ and $x_{2}$. In the design of an experiment approach, the most important goal is to find the outcome or response in a limited number of experiments. As the welding simulations require time and computational cost, they necessitate the use of the design of experiments. The experiments should be designed in such a manner that each contributing factor should have an equal representation. The experiments are arranged in a matrix or table. In designing experiments for the simulation of the welding process, the extreme values of a factor must not be combined in a single experiment; for example, the highest values of current, voltage, and the fastest welding speed must not be combined because that leads to unfeasible solutions. To avoid such unfeasible solutions, in the present work, the design of experiments table was prepared using the Box–Behnken design [33] with three factors, as listed in Table 3. It can be observed from Table 3 that each experiment has at least one mid-range value; for example, in experiment number 4, the welding current and voltage were 80 A and 13 V respectively, which corresponded to their higher value, while the welding speed was 5 mm/s, which was a mid-range value. The responses were the welding-induced residual stress and the distortion produced during welding. The two responses (i.e., residual stress and distortion) were recorded simultaneously during each run of the experiment (i.e., the thermomechanical simulation). The residual stress and distortion were measured on the weld axis.

The last three experiments (i.e., #13, #14, and #15) had identical weld inputs consisting of central values for each parameter. Experiments based on these inputs are called the central run in the Box–Behnken design. The values of the parameters for the central run were obtained using the average of the respective parameter values.

**Figure 4:**
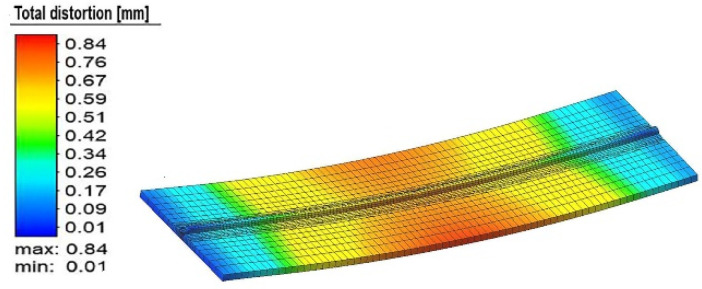
The total distortion in experiment number 3.

The overall distortion observed during the simulation of experimental run #3 is shown in Figure 4. In the figure, the overall distortion of the base metal is shown. This type of distortion is classified as bowing or buckling of the plate. In the butt joint configuration, buckling with a slight amount of twisting or angular distortion was observed. The plate was deformed in the negative z-direction (i.e., along with the thickness).

**Figure 5:**
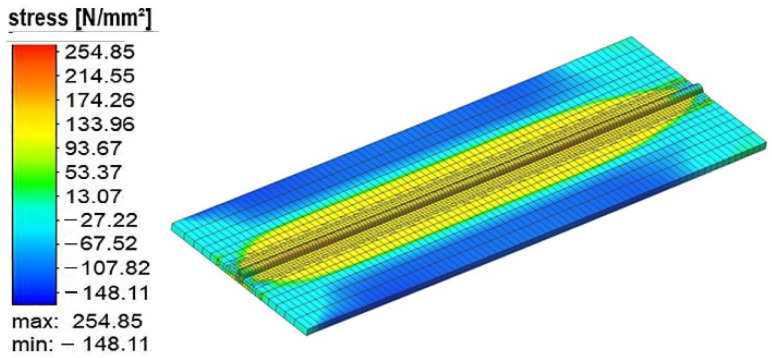
The welding induced residual stress.

In Figure 5, the welding-induced residual stress of experiment run #10 is shown. The residual stress was tensile along with the weld bead. There was compressive residual stress away from the weld centerline.

Through the FEM simulations, the welding residual stress and distortion for all of the experimental runs were similarly calculated. In the next step, the data collected were used to run a regression analysis. Regression analysis was used to find the relation between the weld parameters and their effect on the respective responses (i.e., welding distortion and residual stress). The total welding distortion $R_{1}$ (in mm) and the maximum tensile residual stress $R_{2}$ (in MPa) are expressed in terms of welding current ($X_{1}$), voltage ($X_{2}$), and welding speed ($X_{3}$) in Equations (3) and (4), respectively.

(3) $$R_{1}=32.75-1.197X_{1}+4.486X_{2}-6.035X_{3}+0.006160{X_{1}}^{2}-0.1835{X_{2}}^{2}+0.1690{X_{3}}^{2}+0.00050X_{1}X_{2}+0.058X_{1}X_{3}-0.0075X_{2}X_{3}$$

(4) $$R_{2}=1580-33.28X_{1}-4.7X_{2}-16.5X_{3}+0.1987{X_{1}}^{2}+0.22{X_{2}}^{2}+0.97{X_{3}}^{2}+0.15X_{1}X_{2}+0.3X_{1}X_{3}-1.75X_{2}X_{3}$$

**Figure 6:**
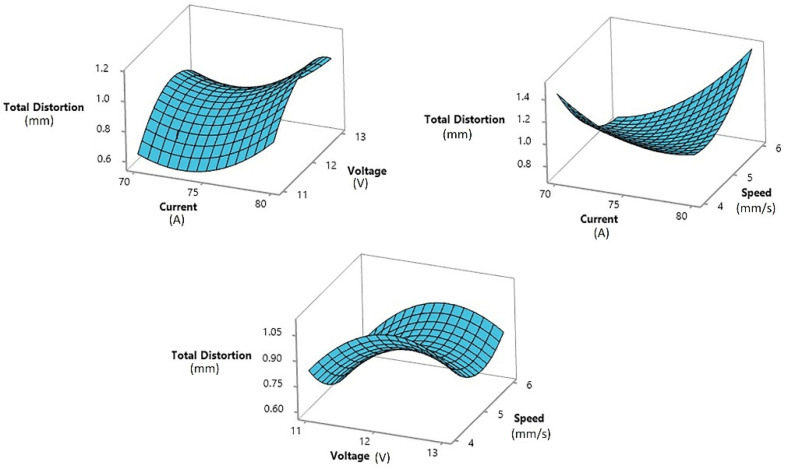
The surface plot of the distortion vs. welding current, voltage, and speed.

Equations (3) and (4) show the contribution of each factor and the combination of their products. A full quadratic analysis was run, which is evident from the quadratic terms in both the equations. The effect of an individual factor on the response variable can be observed in the surface plots. The surface plots of distortion against the three influencing factors are shown in Figure 6.

The surface plot of distortion vs. the welding current and voltage was a saddle-type plot. In this surface plot, the total distortion decreased at the midpoint or 75 A current, but in terms of voltage, the distortion increased at the midpoint voltage of 12 V. In the surface plot of distortion vs. voltage and speed, the same behavior of voltage was observed. The distortion first increased at low voltage; it then increased at the midpoint voltage and again decreased at high voltage. In the same surface plot, the behavior of welding speed could be observed. At low- and high-welding speeds, the distortion value increased, with a minimum distortion value at the midpoint of 5 mm/s.

**Figure 7:**
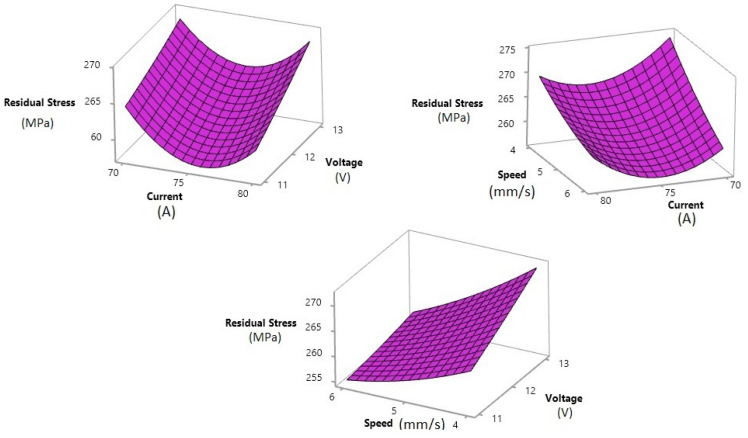
The surface plot of the distortion vs. welding current, voltage, and speed.

The surface plots of residual stress values for the different weld parameters are shown in Figure 7. Welding speed had a different impact on the residual stress. The residual stress decreased with increasing welding speed. In terms of voltage, a lower value of residual stress was observed at minimum voltage. In terms of current, it was the midpoint value of 75 A that produced the minimum residual stress.

### 3.3. Overlaid Contour Plots

**Figure 8:**
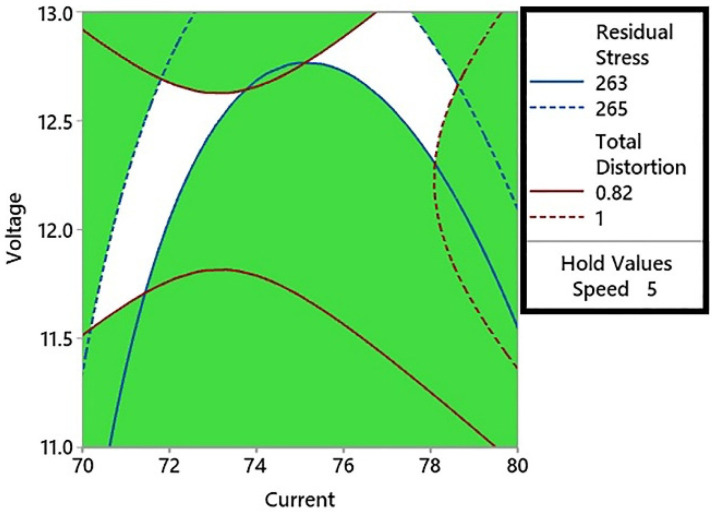
The overlaid contour plot of thee total distortion and residual stress with a speed of 5 mm/s.

Contour plots are the 2D graphical representation of the response or interest to influencing factors on the *x*- and *y*-axis of the plot. In the case of multi-objective optimization studies, two contour plots can be overlaid to find optimum regions for both objective functions. It is a visual technique [34] to find the optimum regions for two or more objectives, but with only two influencing factors. If the number of factors increases to three, the overlaid plots become 3D and hence very difficult to study. An overlaid contour plot for the effect of welding current and voltage on the distortion and residual stress is shown in Figure 8.

**Figure 9:**
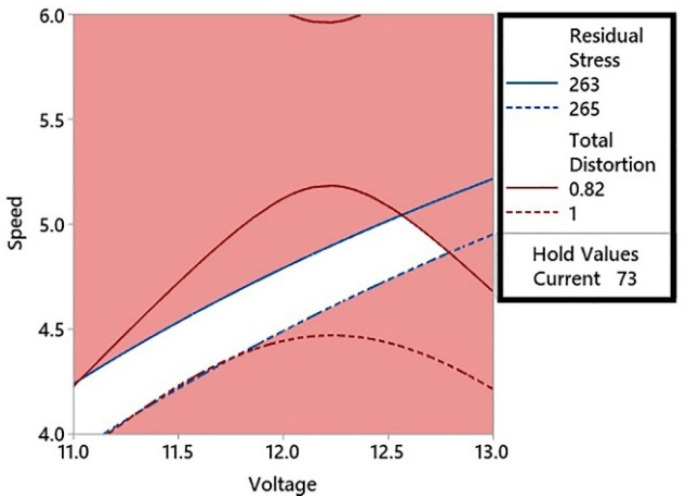
The overlaid contour plot of the total distortion and residual stress with the current at 73 A.

**Figure 10:**
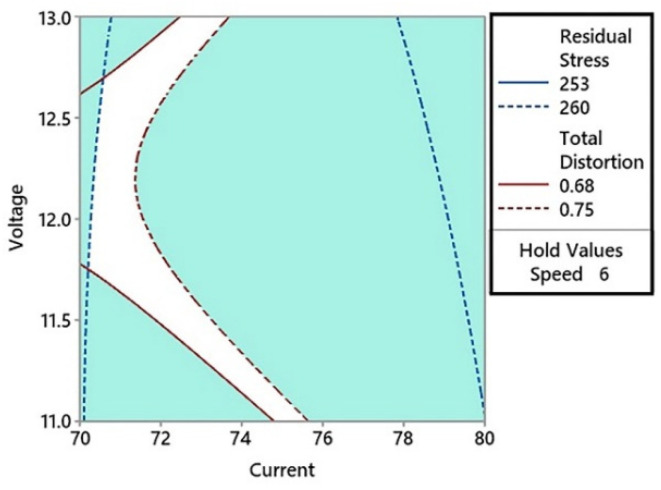
The overlaid contour plot of the total distortion and residual stress with a welding speed of 6 mm/s.

In Figure 8, the residual stress was plotted with an upper limit of 265 MPa and a lower limit of 263 MPa. These values of residual stress represent the minimum residual stress range that intercepts the total distortion range on the welding current and voltage graph. The total distortion values intercepted on the graph were 0.82 mm and 1 mm. It can be observed in the contour plot that the lower value of distortion (0.82 mm) intercepted the lower value of residual stress (263 MPa) at three points. In the left feasible region shown in white, the minimum distortion value (0.82 mm) corresponded to a current value of 73 A. This current value was kept constant and a contour plot was constructed for the welding voltage and speed as shown in Figure 9. These current and voltage values produced the optimum residual stress and distortion in the welded base metal. The advantage of using contour plots is that a range of optimum values for the current, voltage, and welding speed can be obtained. In the surface plots of residual stresses presented in Figure 7, it can be observed that the higher the welding speed, the lower will be the residual stresses produced. To check the optimum range of weld parameters with a combination of higher welding speed, another overlaid contour plot was constructed with the welding speed kept constant at 6 mm/s. This contour plot is shown in Figure 10.

At higher welding speed values, new curve intercepts of welding-induced distortion and residual stress were found. The welding distortion was reduced to 0.68 mm in comparison to the previous minimum value of 0.82 mm. The new optimum value of residual stress was 260 MPa, which is lower than the previous value of 263 MPa. In Figure 10, there was a narrow band of the feasible region. This region corresponded to a range of total distortion (0.68–0.75 mm) and residual stress (253–260 MPa). Several combinations for the welding current and voltage can be selected by picking a point in the optimum region. Starting from the bottom of the contour plot in the middle of the optimum region, the current of 75 A corresponded to a voltage of 11 V. The first feasible set of weld parameters will therefore be 75 A and 11 V at a constant speed of 6 mm/s. A combination of 12 V with 71 A was found in the middle of the *y*-axis. A third combination was found at the top of the contour plot, as shown in Figure 10. This corresponded to the maximum voltage (i.e., 13 V with 73 A of current). It could also be observed from Figure 10 that the feasible region covered the entire range of voltage (from 11–13 V). In the case of current, the feasible range was between 71 A and 75 A.

### 3.4. The Butterfly Optimization Algorithm

The BOA is based on the food searching strategy of butterflies. Capable of differentiating between different fragrances and their intensities, butterflies search for their food source and mating partner by generating and detecting the smell in the air. When a butterfly senses food, it generates a fragrance that can be sensed by other butterflies in the vicinity. In BOA, the intensity of the fragrance is termed as the fitness value of the objective function. The coordinated movement of butterflies toward food is the global search. The butterflies will move randomly if they do not sense any fragrance. This step is defined as the local search. The three phases of the BOA are the initialization phase, the iteration phase, and the final phase. In the initialization phase, the objective function constraints and solution space are defined. In the present work, the solution space has been determined through overlaid contour plots. The initial population of butterflies is also set in the initial phase. The iteration phase is a combination of local and global search. The position of the butterflies changes during this phase. The movement of butterflies is controlled by the number of iterations set during this phase. To switch between the local and global search, a probability p. is defined. In the final phase, a stopping criterion is defined based on the number of iterations. The perceived magnitude of fragrance described by Arora and Singh [29] is presented in Equation (5).

(5) $$\;f=cI^{a}$$

where *f* is the perceived fragrance; I is the stimulus intensity; *c* is the sensory modality; and *a* is the power exponent. *I* is dependent on the objective function. Its value is related to the amount of stimulus generated by a butterfly due to its position in the solution space. How this stimulus is perceived by other butterflies in the solution space is described by *f*. In the actual scenario, there are many physical hindrances such as the direction of air and obstacles between butterflies. These hindrances are interpreted through *c* and *a*. In the present work, the variables and their values used for the butterfly optimization were as follows:

Population size: n=30
Sensory modality: c=0.01
Power exponent: a=0.1
Probability: p=0.8
Number of iterations: N=100

The variable *I* here is a combination of *R*_1_ (welding distortion) calculated through Equation (3) and *R*_2_ (residual stress) calculated through Equation (4). To rationalize the *R*_2_ values, they are divided by 1000. Additionally, the minimization of welding distortion is given a weight of 60% while the minimization of residual stress is given a weight of 40%. The value of *I* is calculated for each butterfly within the solution space. The best solution is selected from all of the available solutions. In the next step, all butterflies move toward the best solution. This movement is initiated after a probability check. For a probability greater than the given value, a global search is initiated; otherwise, a local search is continued. The stopping criterion for the optimization algorithm is the number of iterations, which was set at 100.

## 4. Results

The optimum solution obtained from the BOA is given in Table 4.

**Figure 11:**
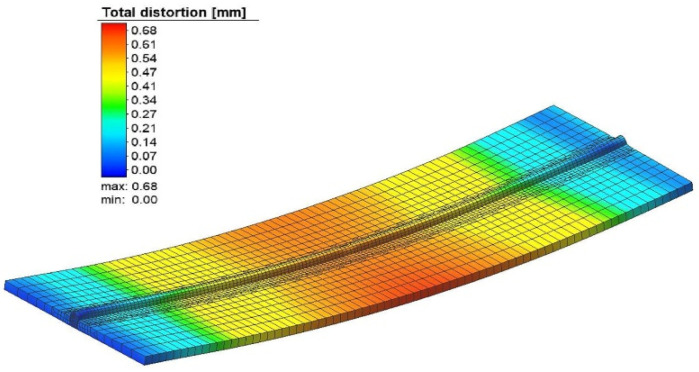
The total distortion at the optimum point.

**Figure 12:**
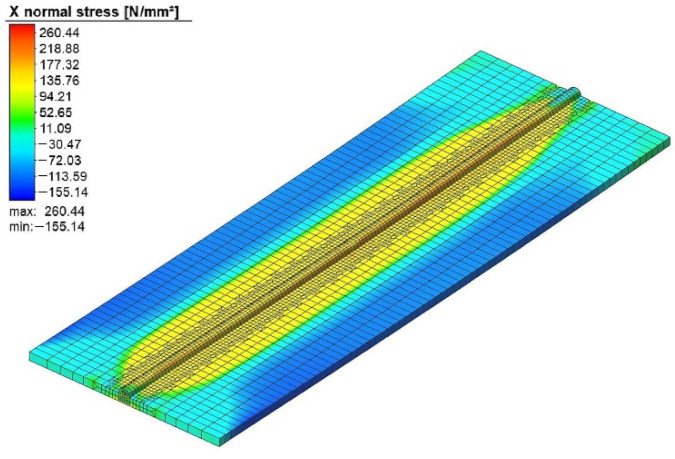
The residual stress at the optimum point.

A welding simulation was performed using these optimum weld parameters to verify that the residual stress and welding-induced distortion fell inside the optimum range. Figure 11 shows the total distortion produced in the optimum solution. The residual stresses produced with the same set of welding parameters are shown in Figure 12.

**Figure 13:**
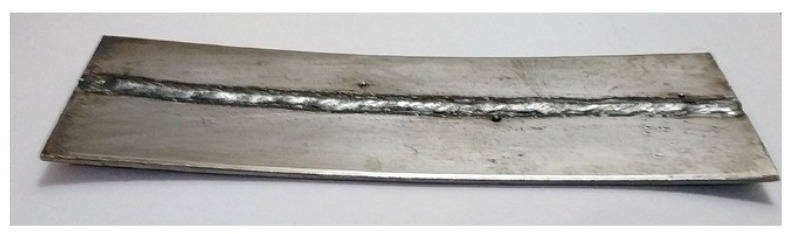
The weld sample for validation.

**Figure 14:**
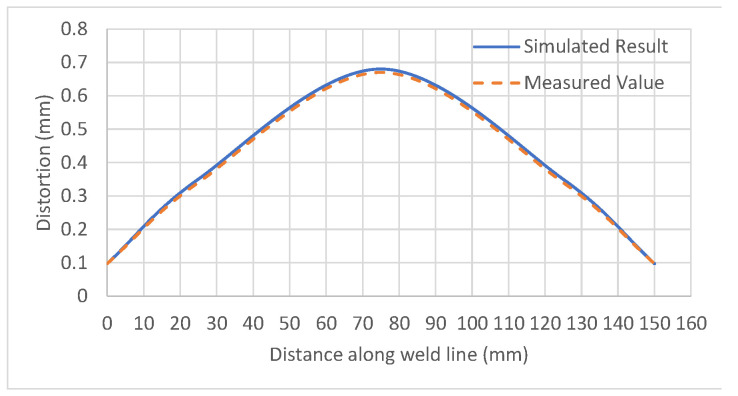
The validation of the distortion results at the optimum point.

To validate the results obtained by the welding simulation, a test sample was prepared by setting the weld parameters of the optimum point of Table 4. The weld sample is shown in Figure 13. The welding-induced distortion was measured along the weld line at 15 equally spaced points. A comparison of the actual and simulated distortion is shown in Figure 14.

In Table 5, the minimum, maximum, and optimum values of both quantities (i.e., distortion and residual stress) are shown.

**Figure 15:**
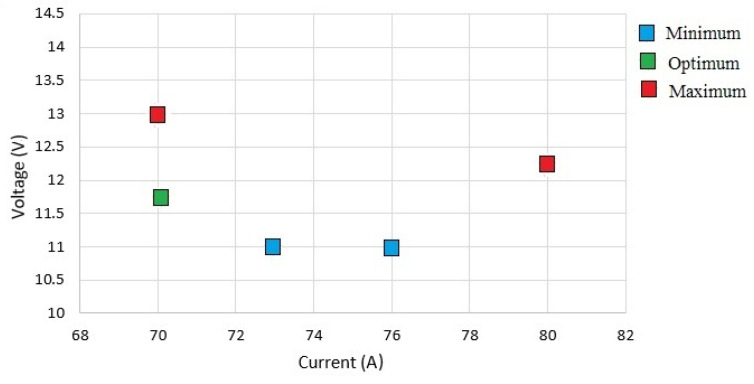
The minimum, optimum, and maximum values of the residual stress and distortion.

It can be concluded from Table 5 that the minimum values of both the residual stress and distortion cannot be achieved at the same values of the welding current and voltage. To obtain the optimum values for both, the welding current was decreased while the voltage was increased to the values shown under ‘Optimum’. The resulting welding-induced distortion and residual stress were plotted against the welding process parameters in Figure 15.

In Figure 15, the blue dots show the minimum values of the residual stress and welding-induced distortion. The values can be verified from Table 5. It should be noted that the minimum values of residual stress and distortion were realized at different values of the welding parameters. The left blue dot represents the welding distortion while the right blue dot represents the residual stress. In a similar manner, red dots show the other extreme (i.e., the maximum values). The green dot represents the optimum values that coincide with each other because they were realized at the same settings of voltage and current.

## 5. Conclusions

A multi-objective study was performed to simultaneously optimize the welding-induced distortion and residual stresses in thin metal plates for a butt joint-weld configuration. The optimum weld parameters to minimize welding-induced distortion and residual stresses were selected by using the response surface method. The response surfaces were utilized to generate a series of contour plots. The contour plots for the minimum distortion and residual stress were overlaid to determine an optimum region. This region contained the values of the optimum weld parameters within a range having upper and lower bounds.

The butterfly optimization algorithm (BOA) was applied to obtain the optimum value of the weld parameters from this solution space. These weld parameters provided the optimum values for the welding-induced distortion and residual stress. In this study, the BOA, as a multi-objective optimization technique, was successfully applied for the first time to a welding problem.

## Figures and Tables

**Table 1:** The chemical composition of ASTM A36.

Carbon (C)	Manganese (Mn)	Phosphorous (P)	Sulfur (S)	Silicon (Si)	Copper (Cu)	Chromium (Cr)	Nickle (Ni)	Molybdenum (Mo)	Vanadium (V)	Titanium (Ti)	Niobium (Nb)	Iron (Fe)
0.15	0.69	0.018	0.004	0.18	0.24	0.15	0.088	0.0195	0.0048	0.0012	0.0024	98.4521

**Table 2:** The double ellipsoidal heat source parameters.

**Double Ellipsoidal** **Heat Source** **Parameter**	**Length** **(mm)**	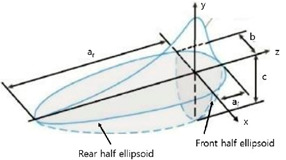
$a_{f}$	2	
$a_{r}$	6	
b	3	
c	2	

**Table 3:** The DOE matrix.

Sr #	Current (A) X_1_	Voltage (V) X_2_	Welding Speed (mm/s) X_3_
1	70	11	5
2	70	13	5
3	80	11	5
4	80	13	5
5	70	12	4
6	70	12	6
7	80	12	4
8	80	12	6
9	75	11	4
10	75	11	6
11	75	13	4
12	75	13	6
13	75	12	5
14	75	12	5
15	75	12	5

**Table 4:** The optimum solution.

	Current (A)	Voltage (V)	Total Distortion (mm)	Residual Stress (MPa)
Optimum Solution	70.2	11.75	0.68	260

**Table 5:** A summary of the results.

	Minimum		Optimum		Maximum	
	Residual Stress 258 MPa	Distortion 0.6 mm	Residual Stress 260 MPa	Distortion 0.68 mm	Residual Stress 275 MPa	Distortion 1.4 mm
Current (A)	76	73	70.2	70.2	70	80
Voltage (V)	11	11	11.75	11.75	13	12.25
